# Clinical and prognostic analysis of 42 children with malignant rhabdoid tumor of the kidney: a 7-year retrospective multi-center study

**DOI:** 10.1186/s12887-022-03643-1

**Published:** 2022-10-13

**Authors:** Chenghao Zhanghuang, Zhaoxia Zhang, Li Zeng, Bing Yan, Haoyu Tang, Jinkui Wang, Xing Liu, Guanghui Wei, Dawei He

**Affiliations:** 1 Department of Urology, Chongqing Key Laboratory of Children Urogenital Development and Tissue Engineering, Chongqing Key Laboratory of Pediatrics, Ministry of Education Key Laboratory of Child Development and Disorders, National Clinical Research Center for Child Health and Disorders, China International Science and Technology Cooperation base of Child development and Critical Disorders, Children’s Hospital of Chongqing Medical University, 2 ZhongShan Rd, 400013 Chongqing, PR China; 2 Department of Urology, Yunnan Provincial Key Research Laboratory of Pediatric Major Diseases, Yunnan Province Clinical Research Center for Children’s Health and Disease, Kunming Children’s Hospital, Children’s Hospital Affiliated to Kunming Medical University, Kunming, PR China; 3 Department of Pediatric Surgical, West China Hospital, Sichuan University, Chengdu, PR China

**Keywords:** Children, Malignant rhabdoid tumor of the kidney, Multi-centers, Prognostic analysis, Diagnosis and treatment

## Abstract

**Objective:**

To discuss the clinical and prognostic indicators of pediatric malignant rhabdoid tumor of the kidney (MRTK), and to increase the understanding of the occurrence and development of MRTK.

**Methods:**

From July 2014 to September 2021, all cases were confirmed by postoperative pathological examination. Among the 42 patients, there were 25 males and 17 females, with a median age of 10 (1–84) months. Abdominal mass or hematuria were the main clinical manifestations. Preoperative chemotherapy was performed in 9 cases (VC). The tumor stages were stage I-IV. Preoperative metastasis was found in 9 cases; the most common site was the lung. Postoperative patients received conventional chemotherapy, including VDACE regimen and UH-1 regimen. Among the 42 children in this group, survival at follow-up in this study was 26.2%(11/42).

**Results:**

Preoperative anemia was found by univariate analysis, hypertension and hypercalcemia had shorter survival time. In addition, tumor-related factors had a significant impact on survival, with incomplete tumor resection, lymph node metastasis, stage III-IV had a lower survival rate. The impact of postoperative factors on survival included postoperative complications had a lower survival rate. The children were younger than 12 months, preoperative metastasis, no chemotherapy was performed after surgery was an independent risk factor for the prognosis of MRTK.

**Conclusion:**

The main clinical manifestations about MRTK were abdominal mass and hematuria. Preoperative chemotherapy did not significantly improve the prognosis. Postoperative chemotherapy can significantly improve the survival rate. Diagnosis depends on clinical manifestations, imaging, histopathology, immunohistochemistry and other comprehensive judgment. Age less than 12 months, preoperative metastasis, and no postoperative chemotherapy were independent risk factors for prognosis.

**Supplementary Information:**

The online version contains supplementary material available at 10.1186/s12887-022-03643-1.

## Introduction

Malignant rhabdoid tumor of the kidney (MRTK) is an embryonal tumor [1, 2] that occurs in the kidney, with an atypical teratoid / rhabdomymatoma (AT/RT) originating from the central nervous system and extrarenal extracranial rhabdomyoid tumor (EERT) originating from the extra-central nervous system, jointly constitute malignant rhabdomyoid tumor (MRT)[3]. MRTK is a highly malignant tumor occurring in the kidney, mostly in infants, especially those under 12 months[1], accounting for about 0.9-2% of pediatric kidney tumors [2]. As MRTK is characterized by high malignancy, high aggressiveness and rapid progression, the prognosis of children is very poor [4]. According to the National Wilms Tumor Study (NWTS) Group, of 142 cases of MRTK collected from 1969 to 2002, the 4-year overall survival (OS) was 23.2%[2]. Brennan et al. reported that among 106 children diagnosed with extracranial MRT in the UK from 1993 to 2010, the 1-year survival rate was only 31%[5]. Therefore, researchers are trying to find better therapeutic measures to improve the survival of children with MRTK. Yamamoto et al. reported that ICE combined with VDCy could achieve a good prognosis in 1 case of stage IV MRTK [6], and Koga et al. treated two patients of MRTK with autologous peripheral blood stem cell transplantation, which achieved long-term survival [7]. In recent years, with the gradual increase of gene studies on MRTK, targeted therapy has become a new direction of research in treating children with MRTK [8].

However, due to the rarity of cases, there are only some scattered reports [9, 10], primarily descriptive studies. Reports on a large-sample statistical analysis of prognostic factors in pediatric MRTK cases are few. Combined with the above situation, to further understand MRTK, extensive sample studies and predictive analysis are critical.

The present study was a multi-center review of 42 pediatric MRTK cases from three large-scale medical centers in southwest China over the past 7 years. In this study, we analyzed the prognostic factors of MRTK from the aspects of clinical characteristics, pathological manifestations, diagnosis and treatment in order to find the prognostic factors of children with MRTK, improve the understanding of the disease and increase the experience of diagnosis and treatment of the disease.

## Methods

### Subjects

A retrospective analysis was performed on 42 children diagnosed with MRTK by pathological examination from Children’s Hospital Affiliated to Chongqing Medical University, West China Medical College of Sichuan University and Kunming Children’s Hospital from July 2014 to September 2021. All cases were confirmed by postoperative pathological examination. Gender, age, primary tumor side, clinical symptoms, laboratory examination (including anaemia, hypertension, hypercalcemia), imaging examination, preoperative chemotherapy (tumor size before and after chemotherapy) and stage were collected. Intraoperative information including tumor rupture, midline crossing, tumor embolism, surrounding tissue invasion, lymph node metastasis, and complete resection were included in this study. In addition, we collected postoperative information including immunohistochemical results, treatment options, and prognostic characteristics. Staging according to the standards set by the Society International Oncology of Pediatric (SIOP) [11], 42 children were divided into stages I-IV. This study was approved by ethical committee of Children’s Hospital of Chongqing Medical University [(2021) Lunshen (Yan) No. (359)]; Children’s Hospital Affiliated to Kunming Medical University (2021-03-002-K01); West China Hospital Affiliated to Sichuan University[2020-shen No. (1062)], and informed consent was obtained from all subjects or their legal guardian(s) / parents (for children’s below 16 years old). And this study is accordance with the relevant guidelines and regulation.

### Treatment protocols

All 42 children underwent physical examination, complete blood, urine routine, blood biochemistry, chest and abdomen CT enhanced examination and blood pressure measurement. According to the World Health Organization’s anemia diagnostic criteria: 1–4 months, hemoglobin (HB) < 90 g/L; 4–6 months, HB < 100 g/L; From 6 months to 5 years old, HB < 110 g/L; From 5 to 11 years old, HB < 115 g/L; Between 12 and 14 years of age, HB < 120 g/L, and anemia was judged.

**Figure 1:**
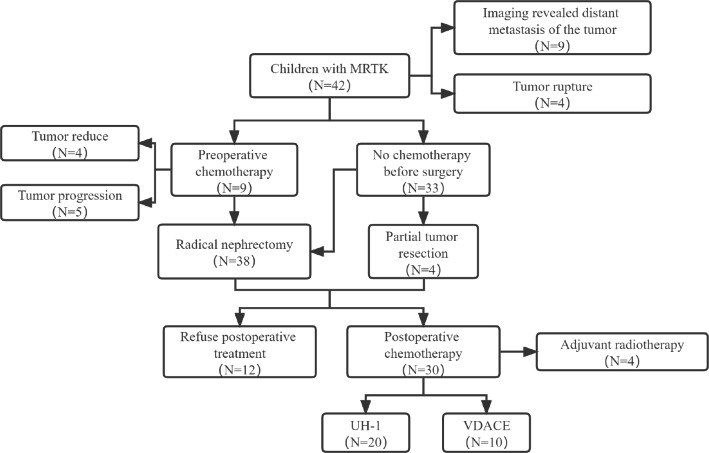
Flowchart of the treatment of MRTK children

There is no clear standard for hypertension in children. This study was based on the diagnostic criteria of cardiology and endocrinology: 0–1 month > 12.0/8.0 kPa(90/60mm Hg), 1 month-3 years > 13.3/8.0 kPa(100/60mm Hg), 3–6 years > 14.7/9.33 kPa(110/70mm Hg). > 14.7/10.7 kPa(110/80mm Hg) in 6–12 years old. And more than three measurements can be diagnosed. The normal value of serum calcium in children is 2.23–2.80 nmol/L. Hypercalcemia was diagnosed when the concentration was higher than 2.80 nmol/L. According to Chinese Children Cancer Group (CCCG) [12] and SIOP guidelines, some children with heavy adhesion and large tumor volume should be given VA regimen before surgery with vincristine (VCR) combined of actinomycin D (ACD). Abdominal CT was re-examined after chemotherapy. According to RECIST guideline [13], the longest single diameter was used as the evaluation standard of tumor size. Surgical methods include needle biopsy, partial tumor resection and radical tumor resection. All the children were examined by pathophysiology and immunohistochemistry. And received chemotherapy followed the UH 1 regimen recommended by CCCG[12], including vincristine, doxorubicin, cyclophosphamide (CTX) combined with cyclophosphamide, carboplatin and etoposide (VP-16) alternated chemotherapy. And VDACE regimen recommended by Pediatric Solid Tumor Diagnosis and Treatment Guidelines, namely vincristin, actinomycin D, doxorubicin, cyclophosphamide and etoposide [14]. Standard, systematic 12 courses of chemotherapy were performed at 1-month intervals. The flowchart is shown in Fig. 1.

### Follow-up analysis

Thoracic and abdominal B-ultrasonography, brain magnetic resonance imaging(MRI), vertebral body and bilateral hip/knee X-ray examinations were reviewed every 3 months within 1 year after surgery. After 1 year, the follow-up was adjusted to every 6 months, and after 3 years, the follow-up was adjusted to every 12 months. Review items are the same as before. The last follow-up was on February 28, 2022.

### Statistical method

All statistical analyses were performed using SPSS 23.0 (IBM, Chicago, IL, USA) and R software (version 3.4.1; http://www.Rproject.org) for data analysis. Measurement data (tumor size, operation time, bleeding) were tested for normality. For data not subject to the normal distribution, median (quartile spacing) was used for description, and the nonparametric test (U test) was used for different analyses. For the data subject to a normal distribution, the mean (standard deviation) was used to describe, and a t-test was used to analyze the difference. For counting data, frequency (%) was used for description, chi-square analysis (including continuity correction method and Fisher’s exact probability method) and nonparametric U test (tumor stage) were used for analysis. Univariate and multivariate Cox regression analyses were performed for all prognostic factors to screen out independent risk factors. The children were divided into groups according to different variables, and the Kaplan-Meier curve and log-rank test were used to analyze differences in patient survival.

## Results

### Clinical characteristics

Among the 42 children with MRTK in this study, there were 25 males and 17 females (sex ratio: 1.5:1), 20 on the left and 22 on the right. The median age of diagnosis was 10 (1–84) months. Abdominal mass was found in 50.0% of the children (21/42) and hematuria was found in 35.7% of the children (15/42). At the same time, 33.3% of the children had anemia (14/42). Other findings included fever (11/42) in 26.2% of patients, hypertension (4/42) and hypercalcemia (4/42) in 9.5%. Tumor metastasis was found in 21.4% of the children (9/42) by imaging examination, including 4 cases of lung metastasis, 3 cases of abdominal and retroperitoneal metastasis, 1 case of spinal bone metastasis, and 1 case of ipsilateral psoas major metastasis. Preoperative CT examination showed that the maximum diameter of the tumor was (9.1 ± 3.9) cm.

### Treatment and prognosis

9 children received chemotherapy before surgery, using VA[12] (Vincristine + Actinomycin D). Among them, 4 patients showed maximum single-path reduction after chemotherapy, and 5 patients showed tumor enlargement. All children underwent surgical treatment, including partial tumor resection and radical resection, under general anesthesia. Radical tumor resection was performed in 88.1% of the patients (38/42). Among them, 1 child underwent preoperative puncture biopsy, and the pathological result was inclined to MRTK, so radical nephrectomy was performed under general anesthesia. 9.5% of the children underwent emergency intraoperative hemostasis and partial tumor resection due to preoperative tumor rupture accompanied by persistent bleeding (4/42). The operative time was (115.1 ± 34.4) min, and the median intraoperative blood loss was 70 (Inter-Quartile range, 20–200) ml. During the operation, 50.0% of the tumors crossed the midline of the abdomen (21/42), 54.8% of the tumors were incomplete (23/42), 19.0% were associated with vena cava tumor thrombus (8/42), 28.6% had surrounding tissue infiltration (12/42), and 11.9% had lymph node metastasis (5/42) as indicated by intraoperative pathology. According to the staging criteria of pediatric renal tumors set by the International Society of Pediatric Oncology (SIOP) [11], the children included in this study were stage I 6 (14.3%), Stage II 5 (11.9%), Stage III 21 (50%), and stage IV 10 (23.8%).

Perioperative complications occurred in 11.9% of the children (5/42), including 2 cases of pleural effusion and 1 case of massive ascites for puncture and drainage, and 1 case of chylous fistula for conservative treatment and recovery after parenteral nutrition. A case of intractable hypertension was transferred to endocrinology department for drug control after multidisciplinary diagnosis and treatment (MDT). 28.6% of the children were discharged from hospital after family members refused treatment (12/42). Standardized postoperative chemotherapy was received in 71.4% of the patients (30/42), including 20 patients using UH-1 and 10 patients using VDACE. Only 4 patients received postoperative radiotherapy.

**Figure 2:**
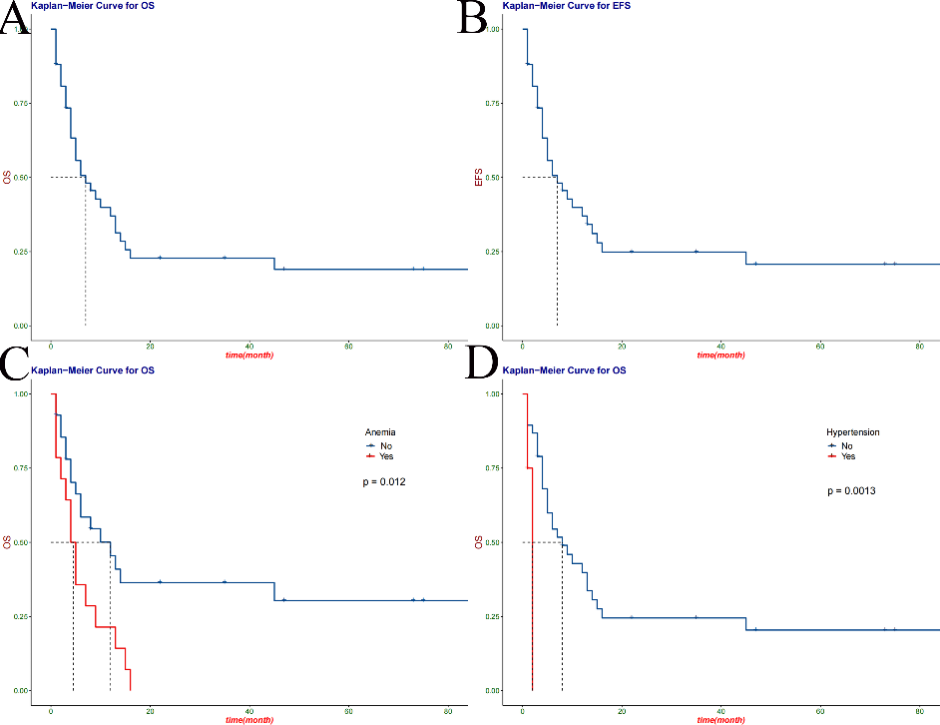
Kaplan-meier curves for overall or event-free survival in patients with OS(**A**), EFS(**B**), anemia (**C**), and hypertension (**D**)

In this study, 42 children were followed up from 2 months to 86 months, with a median follow-up of 5 months. The survival at follow-up in this study was 26.2% (11/42). Among the 31 deaths, 96.8% were due to tumor recurrence and metastasis (30/31), and 3.2% were due to systemic septic shock caused by toxic side effects of chemotherapy drugs (1/31). In this study, the 1-year, 3-year and 5-year overall survival rates (OS) of children with MRTK were 37.0% (95%CI: 0.245–0.559), 24.9% (95%CI: 0.140–0.440) and 20.7% (95%CI: 0.106–0.406). The event free survival (EFS) were 37.0% (95%CI: 0.2451–0.559), 22.8% (95%CI: 0.1256–0.413) and 19.0% (95%CI: 0.0948-0.380) (Fig. 2 A-B). Relevant data are shown in Table 1.

**Table 1:** Clinicopathological characteristics of patients with MRTK.

	Total	Alive	Dead	P
	42	11	31	
Sex (%)				
1	25 (59.52)	6 (54.55)	19 (61.29)	0.9728
2	17 (40.48)	5 (45.45)	12 (38.71)	
Age (%)				
≤ 12 months	22 (52.38)	3 (27.27)	19 (61.29)	0.112
> 12 months	20 (47.62)	8 (72.73)	12 (38.71)	
Metastasis at diagnosis (%)				
No	33 (78.57)	11 (100.00)	22 (70.97)	0.1122
Yes	9 (21.43)	0 (0.00)	9 (29.03)	
Fever (%)				
No	31 (73.81)	9 (81.82)	22 (70.97)	0.7611
Yes	11 (26.19)	2 (18.18)	9 (29.03)	
Anemia (%)				
No	28 (66.67)	11 (100.00)	17 (54.84)	0.0184
Yes	14 (33.33)	0 (0.00)	14 (45.16)	
Hematuresis (%)				
No	27 (64.29)	7 (63.64)	20 (64.52)	1
Yes	15 (35.71)	4 (36.36)	11 (35.48)	
Hypertension (%)				
No	38 (90.48)	10 (90.91)	28 (90.32)	1
Yes	4 (9.52)	1 (9.09)	3 (9.68)	
Hypercalcemia (%)				
No	38 (90.48)	11 (100.00)	27 (87.10)	0.5127
Yes	4 (9.52)	0 (0.00)	4 (12.90)	
Preoperative chemotherapy (%)				
No	33 (78.57)	10 (90.91)	23 (74.19)	0.4635
Yes	9 (21.43)	1 (9.09)	8 (25.81)	
Tumor size (mean (SD))	9.112 (3.908)	8.518 (4.622)	9.323 (3.684)	0.564
Operation time (mean (SD))	115.071 (34.384)	121.273 (37.227)	112.871 (33.686)	0.4931
Hemorrhage (median [IQR])	70 [20, 200]	35 [20, 110]	100[35, 200]	0.172
Tumor location (%)				
Full	9 (21.43)	1 (9.09)	8 (25.81)	0.0131
Upper	16 (38.10)	5 (45.45)	11 (35.48)	
Middle	14 (33.33)	2 (18.18)	12 (38.71)	
Lower	3 (7.14)	3 (27.27)	0 (0.00)	
Tumor side (%)				
Left	22 (52.38)	7 (63.64)	15 (48.39)	0.604
Right	20 (47.62)	4 (36.36)	16 (51.61)	
Cross the center line (%)				
No	21 (50.00)	4 (36.36)	17 (54.84)	0.4827
Yes	21 (50.00)	7 (63.64)	14 (45.16)	
Tumor rupture (%)				
No	27 (64.29)	8 (72.73)	19 (61.29)	0.7536
Yes	15 (35.71)	3 (27.27)	12 (38.71)	
Capsule (%)				
No	19 (45.24)	8 (72.73)	11 (35.48)	0.0751
Yes	23 (54.76)	3 (27.27)	20 (64.52)	
Incomplete tumor resection (%)				
No	36 (85.71)	11 (100.00)	25 (80.65)	0.2826
Yes	6 (14.29)	0 (0.00)	6 (19.35)	
Tumor embolus (%)				
No	34 (80.95)	9 (81.82)	25 (80.65)	1
Yes	8 (19.05)	2 (18.18)	6 (19.35)	
Infiltration (%)				
No	30 (71.43)	9 (81.82)	21 (67.74)	0.6175
Yes	12 (28.57)	2 (18.18)	10 (32.26)	
Lymph node metastasis (%)				
No	37 (88.10)	11 (100.00)	26 (83.87)	0.3803
Yes	5 (11.90)	0 (0.00)	5 (16.13)	
Tumor stage (%)				
I	6 (14.29)	4 (36.36)	2 (6.45)	0.0269
II	5 (11.90)	2 (18.18)	3 (9.68)	
III	21 (50.00)	5 (45.45)	16 (51.61)	
IV	10 (23.81)	0 (0.00)	10 (32.26)	
Preoperative chemotherapy (%)				
No	30 (71.43)	9 (81.82)	21 (67.74)	0.6175
Yes	12 (28.57)	2 (18.18)	10 (32.26)	
Chemotherapy protocol (%)				
No	12 (28.57)	2 (18.18)	10 (32.26)	0.6743
VDACE	10 (23.81)	3 (27.27)	7 (22.58)	
HU-1	20 (47.62)	6 (54.55)	14 (45.16)	
Preoperative metastasis (%)				
No	18 (42.86)	11 (100.00)	7 (22.58)	< 0.0001
Yes	24 (57.14)	0 (0.00)	24 (77.42)	

**Figure 3:**
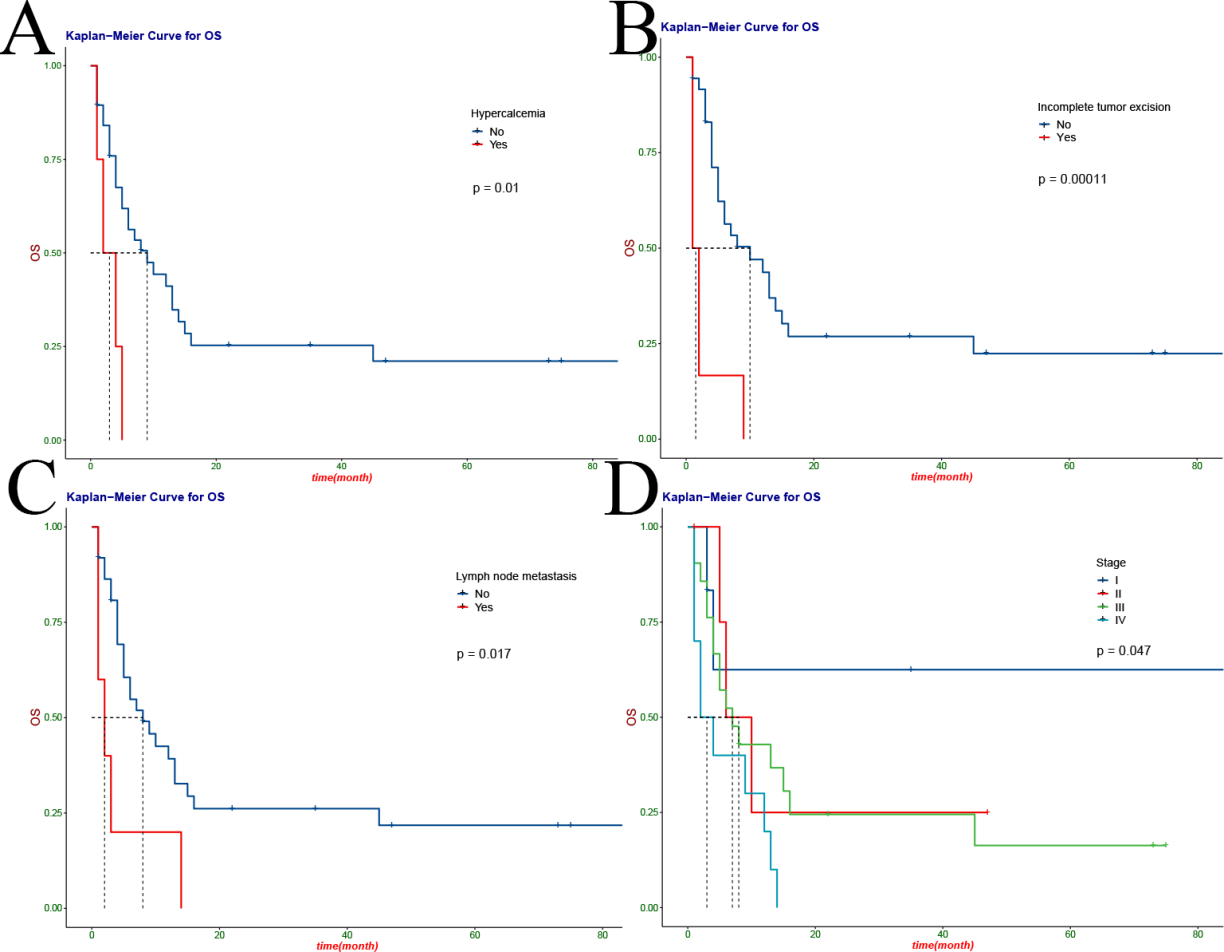
Kaplan-meier curve of overall survival in children with MRTK: hypercalcaemia (**A**), incomplete tumor resection (**B**), lymph node metastasis (**C**) and tumor stage (**D**)

**Figure 4:**
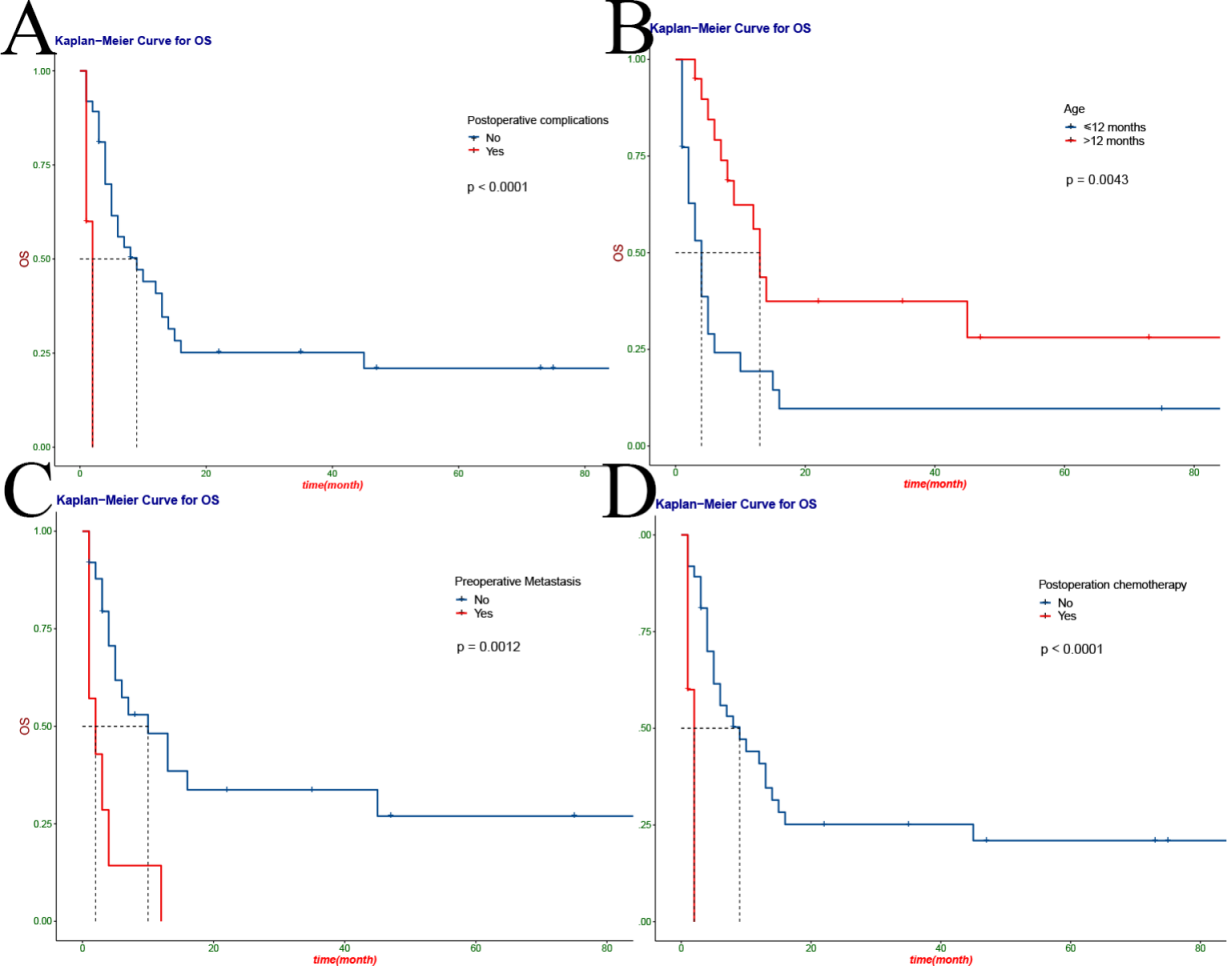
Kaplan-Meier curves for overall survival of children with MRTK, patients according to postoperative complications(**A**), age(**B**), preoperative metastasis(**C**) and postoperative chemotherapy(**D**)

We performed a univariate analysis of all variables and found preoperative anaemia (HR 2.47, 95%CI: 1.2–5.11; P = 0.012), hypertension (HR 8.45, 95%CI: 1.94–36.82; P = 0.0013) and hypercalcemia (HR 4.15, 95%CI: 1.35–12.79; P = 0.01) (Fig. 2 C-D, Fig. 3 A) had shorter survival time. In addition, tumor factors had a significant impact on survival, with incomplete tumor resection (HR 5.73, 95%CI: 2.2-14.92; P = 0.00011), lymph node metastasis (HR 3.18, 95%CI: 1.19–8.45; P = 0.017), tumor stage (HR, 1.77, 95%CI: 1.12–2.8; P = 0.047) had a lower survival rate (Fig. 3B-D). The influence of postoperative factors on survival mainly included postoperative complications (HR 13.09, 95%CI: 3.07–55.78; P<0.0001), which had a lower survival rate (Fig. 4 A). The children were older than 12 months (HR 0.274, 95%CI: 0.122–0.617; P = 0.0043), preoperative metastasis (HR 4.23, 95%CI: 1.649–10.85; P = 0.0012), no postoperative chemotherapy (HR 3.155, 95%CI 1.216–8.185; P<0.0001) was an independent risk factor for the prognosis of MRTK (Fig. 4B-D) (Table 2). At the same time, we analyzed the clinical characteristics of operative time, intraoperative bleeding, intraoperative tumor rupture and metastasis for children with or without preoperative chemotherapy. We found that preoperative chemotherapy could not effectively reduce intraoperative bleeding and operative time, nor could it reduce the risk of tumor rupture and metastasis (Table S1).

**Table 2:** Univariate and multivariate analyses in children with MRTK

	Univariate				Multivariate		
	HR	95% CI	P		HR	95% CI	P
Sex	0.89	0.43–1.84	0.753				
Age	0.35	0.17–0.74	0.005		0.274	0.122–0.617	0.0043
Preoperative metastasis	3.47	1.55–7.75	0.002		4.23	1.649–10.85	0.0012
Fever	1.04	0.48–2.27	0.918				
Anemia	2.47	1.2–5.11	0.012				
Hematuresis	0.67	0.31–1.41	0.288				
Hypertension	8.45	1.94–36.82	0.0013				
Hypercalcemia	4.15	1.35–12.79	0.01				
Preoperative chemotherapy	1.32	0.59–2.96	0.497				
Tumor size	1.01	0.92–1.11	0.809				
Operation time	1	0.99–1.01	0.818				
Hemorrhage	1	1–1	0.25				
Tumor site	1.99	0.97–4.09	0.06				
Tumor location	0.62	0.39–0.98	0.042				
Cross the center line	0.58	0.28–1.18	0.131				
Tumor rupture	0.94	0.45–1.95	0.866				
Capsule	1.91	0.91–3.99	0.087				
Incomplete tumor resection	5.73	2.2-14.92	0.00011				
Tumor embolus	2.21	0.86–5.68	0.099				
Infiltration	1.61	0.74–3.52	0.232				
Lymph node metastasis	3.18	1.19–8.45	0.017				
Tumor stage	1.77	1.12–2.8	0.047				
Postoperative complication	13.09	3.07–55.78	<0.0001				
Postoperative chemotherapy	3.86	1.72–8.67	0.001		3.155	1.216–8.185	<0.0001
Chemotherapy protocol	0.57	0.35–0.91	0.019				

### Immunohistochemical characteristics

All the children underwent postoperative histopathological immunohistochemical tests, and the typical microscopic manifestation of MRTK was that intracytoplasmic eosinophilic inclusion bodies were detected only in 18.8% of the patients (8/42). Due to the large number of immunohistochemical items and the large differences between individuals, we listed the test items covering more than 60% of the children (Table S2). Univariate prognostic correlation analysis was performed on the above collected immunohistochemical data, and it was found that more than 60% of ki-67 positive areas were associated with poor prognosis (HR 2.65, 95%CI: 1.15–6.09; P = 0.021) (Table S3).

## Discussion

MRTK is a rare and highly malignant renal tumor, which belongs to the category of MRT, mainly occurring in children and occurring within 12 months of age [1]. In this study, the sex ratio of MRTK children was 1.5:1, and the median age of diagnosis was 10 (1–84) months. The children were younger than 12 months is an independent risk factor for the prognosis of MRTK, which is consistent with reports of large samples related to MRTK [5, 15, 16]. In addition, when patients with stage I-IV were included in the prognostic analysis, tumor stage was associated with prognosis. However, tumor stage was not an independent prognostic factor for MRTK after we included it in the multifactorial analysis. This is related to the high mortality of the cases included in this study.

In this study, the main clinical manifestations of MRTK children were abdominal mass or hematuria, and other manifestations included fever, hypertension and hypercalcemia. Anemia before surgery, hypertension and hypercalcemia suggested poor prognosis and preoperative metastasis was an independent risk factor for MRTK. Hypertension and hypercalcemia have rarely been reported in previous studies on MRTK, and are considered to be related to paraneoplastic syndrome caused by cancer [17]. Heuvel-eibrink et al. reported that the incidence was 11% and 19%, respectively [18]. There are two main causes of hypertension. Tumor is often a systemic disease, which can not only invade local areas, but also involve various systems of the body. Firstly, the tumor locally compresses the renal artery and its branches, activates the renin-angiotensin-aldosterone system, causes peripheral vascular contraction, water and sodium retention, and leads to hypertension. On the other hand, abnormal coagulation function and intravascular tumor thrombus can also cause the increase of blood pressure to varying degrees [19]. Moreover, disorders of systemic humoral regulatory system and intravascular tumor thrombus are associated with poor prognosis[20]. Renal tumors are prone to hypercalcemia due to the involvement of the kidney in calcium metabolism and maintenance of normal hematocrit[15]. Excessive bone resorption is an important cause of hypercalcemia. Bone metastasis of malignant tumors and tumor-derived humoral mediators are stimulating factors of bone resorption, such as prostaglandin E2, osteoclast activating factor and parathyroid hormone-like polypeptide secreted by tumors can cause increased bone resorption and increase serum calcium[16]. However, there are tumors that do not develop bone metastases and that simultaneously develop an increase in serum calcium levels. This symptom is associated with hormonal peptides secreted by kidney tumor cells and suggests a poor prognosis[21]. In addition, in malignant solid tumors such as bladder cancer, prostate cancer, breast cancer, thyroid cancer and renal cell cancer, distant metastasis has been written into the clinical diagnosis and treatment guidelines of the AJCC as an independent risk factor for prognosis[22]. Our study found that MRTK as a rare renal tumor type, preoperative detection of distant metastasis similarly predicted a worse prognosis. However, due to the small sample size, further data collection is needed to clarify its predictive value for prospective studies.

**Figure 5:**
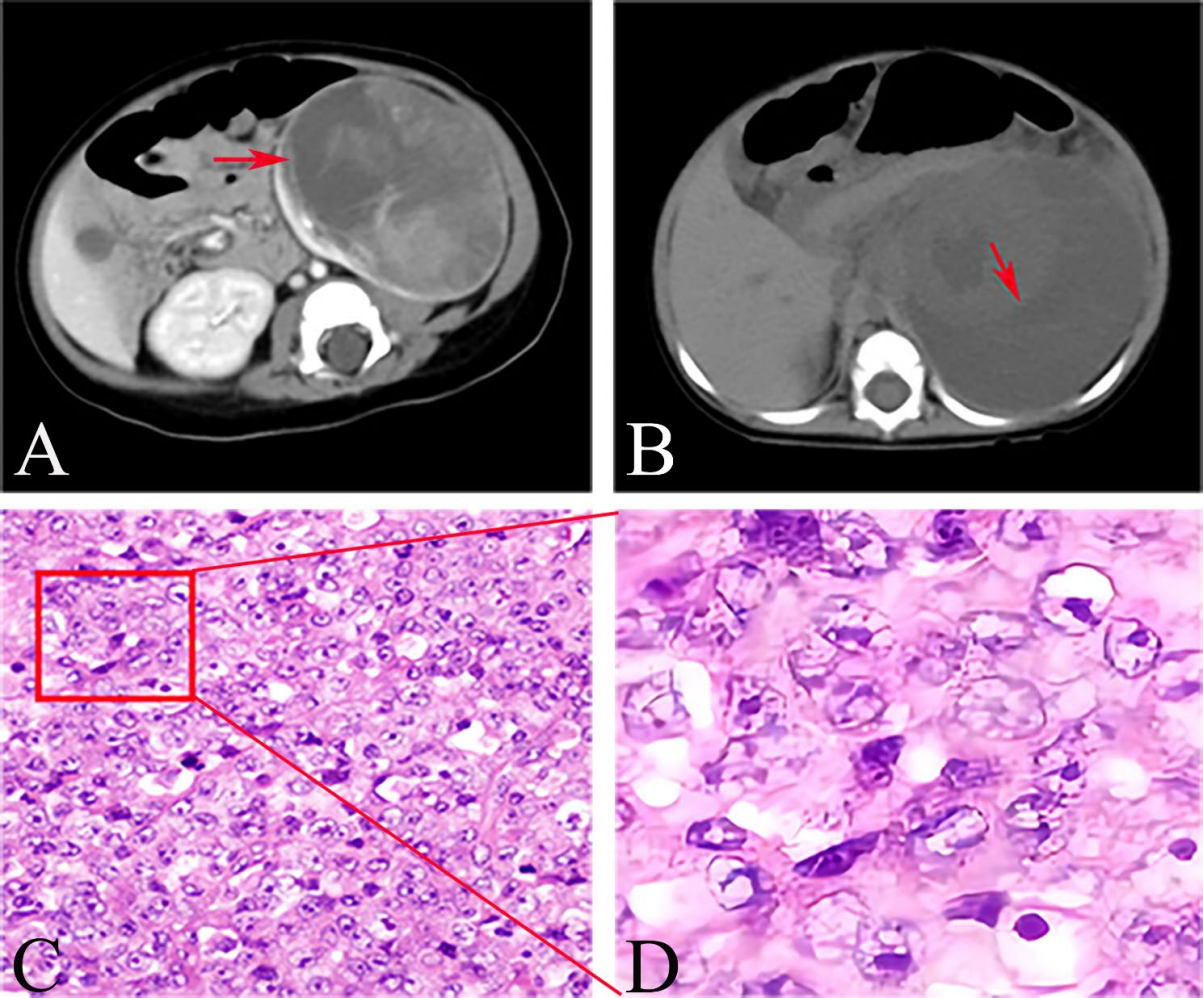
Typical radiological and histopathological changes in children with MRTK. CT scan of the abdomen shows “crescent-shaped” fluid dark area(red arrow) (**A**-**B**).The histopathological findings were microscopic vacuolar nuclei, central eosinophilic nucleoli and intracytoplasmic eosinophilic inclusions.(HE 400×)(**C**),Typical microscopic changes can be seen after magnification(**D**)

In addition, we found that the number of patients with deep vein tumor thrombus was high (8/42, 19%) in this study. We speculate that this is because MRTK and other malignant tumors have a strong ability to invade blood vessels and easily penetrate the blood vessel wall, leading to the occurrence of hematuria and anemia, which then activate the coagulation factor pathway and enhance platelet increase and adhesion. In addition, CT revealed a “crescent-shaped” fluid dark area around the tumor in 55.3% of patients (21/38), except for 4 patients with preoperative tumor rupture (Fig. 5 A-B). We suspect that this is closely related to the aggressive ability of the tumor. Previous studies on MRTK only carried out descriptive analysis of clinical characteristics, but this study included them into prognostic factors for analysis, providing a preliminary theoretical basis for judging prognosis according to clinical manifestations.

In the past decade, the importance of ki-67 in the prognosis of breast cancer has been extensively studied, but there has been little research and literature on renal tumors. Menon et al. emphasized that ki-67 expression is significantly correlated with renal tumor staging and metastasis potential [23]. Li et al. also found that ki-67 was a prognostic risk factor of more than 70% in the clinical study of 14 children with MRTK [24]. In this study, we performed prognostic analysis of immunohistochemical indicators covering more than 24 cases (60%), including INI-1, Vimentin, CK, EMA, Desmin, WT-1, Ki-67, Myogenin, myoD1, Bcl-2, and CyclinD1. More than 60% of ki-67 positive concentrations were found suggesting poor prognosis. There was no significant correlation between other indicators and prognosis. In conclusion, the diagnosis of MRTK needs to be based on clinical manifestations, imaging, histopathology(Fig. 5 C-D), immunohistochemistry and other results, combined with experienced clinicians and pathologists.

Preoperative Fine needle aspiration biopsy (FNAB) is not recommended for definitive diagnosis [17]. First, the positive rate of FNAB is low and target cells cannot be obtained by biopsy to areas such as liquefaction necrosis. At the same time, MRTK tumor tissue is prone to rupture [14], and puncture will cause extrusion and invasive operation to the tumor, increasing the risk of tumor rupture and bleeding. More importantly, FNAB delays surgical treatment. Notably, this study found that complete tumor resection was not an independent risk factor for the prognosis of MRTK. The reason may be related to the high degree of malignancy of MRTK, which is consistent with the conclusion of Heuvel et al. [18]. However, there are still controversies about whether to perform preoperative chemotherapy and postoperative high-dose chemotherapy and radiotherapy. In this paper, the above three controversies are analyzed and discussed in combination with multi-center data.

First, can preoperative chemotherapy improve outcomes in children with MRTK? According to most opinions, preoperative chemotherapy is indispensable for the treatment of MRTK. Schmidt believes that preoperative chemotherapy can effectively reduce tumor volume, reduce the risk of tumor rupture, and reduce the operation time and intraoperative bleeding [25]. Tournade also advocated preoperative chemotherapy as an indispensable and important part of MRTK treatment [26]. However, in this study, (9/42, 21.4%) children received preoperative chemotherapy. The chemotherapy regimen was VA. Preoperative chemotherapy was more effective in reducing tumor volume (4/9 vs. 5/9; P = 0.376) and reduced the risk of tumor rupture (11/33 vs. 4/9; P = 0.8226), reduced operation time [(111.7 ± 33.3) min vs. (127.6 ± 37.2) min; P = 0.2234)] and reduced intraoperative bleeding [50 (Inter-Quartile range, 20–200) ml vs. 100 (Inter-Quartile range, 60–300) ml, P = 0.1392]. Therefore, our study concluded that preoperative chemotherapy did not show a significant therapeutic advantage for children with MRTK.

Second, is the chemotherapy dose as high as possible? Schaff et al. found that the prognosis of primary central nervous system lymphoma (PCNSL) has improved significantly with the introduction and widespread use of high-dose methotrexate (MTX) chemotherapy in the past few decades [27]. Pierantoni demonstrated the effectiveness of high-dose chemotherapy in patients with recurrent germ cell tumor (GCT) [28]. Although it has been agreed that postoperative chemotherapy is necessary for MRTK [17], due to the low incidence and high mortality of MRTK, no large-sample prospective randomized controlled clinical study has been published, and there is no unified standard for postoperative chemotherapy. Currently, internationally, chemotherapy schemes reported by UK-WT [18], GPOH[17], NWTSG[1], SIOP[18] and other authoritative institutions are the mainstream. In this study, UH-1[12] and VDACE[13] regimens were used, and 1-year, 3-year, and 5-year overall survival rates (OS) were 37.0% (95%CI: 0.245–0.559), 24.9% (95%CI: 0.140–0.440), and 20.7% (95%CI: 0.106–0.406). The event free survival (EFS) was 37.0% (95%CI: 0.2451–0.559), 22.8% (95%CI: 0.1256–0.413) and 19.0% (95%CI: 0.0948-0.380). Compared with mainstream schemes in Europe and America, OS showed no significant difference, which was slightly lower in this study.

High dose chemotherapy (HDC) is widely used in modern times because it can significantly improve the survival rate of some malignant tumors. Although postoperative chemotherapy improved prognosis, but the dose and cumulative effect of chemotherapy drugs deserve attention. Zaky reported that 5/19 children with MRTK died due to the toxic and side effects of drugs after high-dose chemotherapy [29]. In this study, 1 child underwent septic shock after bone marrow transplantation after 6 courses of VDACE regimen and eventually died of respiratory failure.

Thirdly, whether to use radiotherapy after surgery and the advantages and disadvantages of high-dose radiotherapy. In response to the low survival rate of MRTK, Reinhard’s 5-year OS increased from 11 to 25% after radiotherapy [30]. Tomlinson improved the 4-year OS of 142 children with MRTK from 12 to 28.5% by radiotherapy [2]. It is gradually accepted that high-dose radiotherapy can improve the prognosis of children with MRTK. However, SIOP proposed 20 years ago that radiotherapy dose should be gradually reduced [31]. Furtwangler also argued that high-dose radiotherapy not only did not improve survival, but increased non-cancer mortality [26]. In addition, radiation is irreversible to the brain and parenchymal tissue damage of infants, so it is considered that infants should not use radiotherapy [32]. In this study, due to various factors (economic factors, toxic and side effects, and young age), only 4 children received chemotherapy combined with radiotherapy. It is noteworthy that all the 4 patients who received postoperative radiotherapy survived. Therefore, we speculated that for children with MRTK older than 2 years, conventional dose of chemotherapy and radiotherapy can effectively improve the prognosis. The three cases collected in this study are located in the mountainous region of southwest China, with backward economic development. When the patients communicated with their family members about the prognosis of MRTK after diagnosis, 90.5% of the family members refused radiotherapy (38/42). Even 28.6% of family members refused chemotherapy (12/42). This is also one of the important reasons why OS in this study is lower than that in some developed countries such as Europe and America [1, 5, 15]. Since only 9.6% of children with MRTK received radiotherapy (4/42), whether it can improve the survival rate of MRTK needs further study.

In recent years, gene targeted therapy has become a new research direction for the treatment of MRTK. Some drugs, such as Ribociclib, a cell cycle gene CDK4/6 inhibitor, Viliparib, a DNA level RARP inhibitor, and LY3023414, a mTOR signaling pathway inhibitor, have entered phase II clinical trials [4]. In addition, our previous study found that PI3K-AKT-mTORC1 signaling pathway may play a key role in the development of MRTK [33]. Moreover, we found that P4HA1, AURKA, GOT1 and MLLT11 are expected to become new targets for the diagnosis and treatment of MRTK [34]. However, the efficacy and safety of treatment still need to be confirmed by international cooperation and large sample prospective studies.

There are still some limitations to this study. First of all, this study is retrospective, and selection bias is unavoidable. Second, this study collected case data from three large children’s medical centers, with slight differences in treatment details such as chemotherapy dose. Third, we lack data such as genetic testing. However, considering the scarce number of MRTK cases, we adopted a long time span, multi-center study to provide help for clinical diagnosis and treatment of MRTK by statistical analysis.

## Conclusion

Retrospective analysis of 42 children with MRTK collected from three large-scale children’s medical centers in southwest China for 7 years shows that MRTK is a highly malignant tumor occurring in the kidney. The clinical manifestations are mainly abdominal mass and hematuria. Preoperative chemotherapy can reduce some tumor volume. However, it cannot effectively improve the prognosis of children with MRTK. Diagnosis depends on clinical manifestations, imaging, histopathology, immunohistochemistry and other comprehensive judgment. Surgery is the main treatment for MRTK, and complete tumor resection should be achieved during the operation. Postoperative chemotherapy can effectively improve the prognosis, and the effect of combined radiotherapy needs to be further studied. Preoperative hypercalcemia, hypertension, tumor rupture, pathological examination showed that the concentration of Ki-67 was greater than 60%, suggesting a poor prognosis. Age at diagnosis less than 12 months, preoperative metastasis, and no chemotherapy were independent risk factors for prognosis.

## Electronic supplementary material

Below is the link to the electronic supplementary material.

Supplementary Material 1

Supplementary Material 2

Supplementary Material 3

## Data Availability

The datasets generated and analysed during the current study are not publicly available due further literature analysis and paper writing will be carried out, but are available from the corresponding author on reasonable request.

## Code Availability

N/A.

## Declarations

This study is accordance with the relevant guidelines and regulation.
