# Supplementary material for: Clinical and prognostic analysis of 42 children with malignant rhabdoid tumor of the kidney: a 7-year retrospective multi-center study
Source: BMC Pediatr. 2022 Oct 13;22:591. doi: 10.1186/s12887-022-03643-1 (PMC9563785; doi:10.1186/s12887-022-03643-1)
Supplement: Supplementary file 1 — Supplementary Material 1 [file 12887_2022_3643_MOESM1_ESM.docx]

Table S1. Clinicopathological characteristics of patients with or without preoperative chemotherapy

|  | Total | No | Yes | p |
| --- | --- | --- | --- | --- |
|  | 42 | 33 | 9 |  |
| Operation time (mean (SD)) | 115.071 (34.384) | 111.667 (33.367) | 127.556 (37.182) | 0.2234 |
| Hemorrhage (median [IQR]) | 70 [20, 200] | 50 [20, 200] | 100 [60, 300] | 0.1392 |
| Tumor rupture (%) |  |  |  |  |
| No | 27 (64.29) | 22 (66.67) | 5 (55.56) | 0.8226 |
| Yes | 15 (35.71) | 11 (33.33) | 4 (44.44) |  |
| Metastasis (%) |  |  |  |  |
| No | 18 (42.86) | 15 (45.45) | 3 (33.33) | 0.7861 |
| Yes | 24 (57.14) | 18 (54.55) | 6 (66.67) |  |
