# Supplementary material for: Clinical and prognostic analysis of 42 children with malignant rhabdoid tumor of the kidney: a 7-year retrospective multi-center study
Source: BMC Pediatr. 2022 Oct 13;22:591. doi: 10.1186/s12887-022-03643-1 (PMC9563785; doi:10.1186/s12887-022-03643-1)
Supplement: Supplementary file 2 — Supplementary Material 2 [file 12887_2022_3643_MOESM2_ESM.docx]

Table S3. Univariate analysis of immunohistochemistry of patients with MRTK

|  | HR | CI | P |
| --- | --- | --- | --- |
| INI_1 | NA | NA-NA | NA |
| Vimentin | NA | NA-NA | NA |
| CK | 0.92 | 0.31-2.73 | 0.882 |
| EMA | 1.28 | 0.38-4.33 | 0.692 |
| Desmin | 2.36 | 0.79-7.05 | 0.124 |
| WT_1 | 1.81 | 0.7-4.7 | 0.221 |
| **Ki_67** | 2.65 | 1.15-6.09 | **0.021** |
| myogenin | 0 | 0-Inf | 0.998 |
| myoD1 | NA | NA-NA | NA |
| Bcl_2 | 1.14 | 0.44-2.9 | 0.791 |
| CyclinD1 | 0.58 | 0.23-1.51 | 0.267 |
