# Supplementary material for: Clinical and prognostic analysis of 42 children with malignant rhabdoid tumor of the kidney: a 7-year retrospective multi-center study
Source: BMC Pediatr. 2022 Oct 13;22:591. doi: 10.1186/s12887-022-03643-1 (PMC9563785; doi:10.1186/s12887-022-03643-1)
Supplement: Supplementary file 3 — Supplementary Material 3 [file 12887_2022_3643_MOESM3_ESM.docx]

Table S2. Immunohistochemistry of patients with MRTK

|  | Overall | Alive | Dead | p |
| --- | --- | --- | --- | --- |
|  | N=42 | N=11 | N=31 |  |
| INI_1 (%) |  |  |  |  |
| (-) | 42 (100.00) | 11 (100.00) | 31(100.00) | NA |
| Vimentin (%) |  |  |  |  |
| (+) | 32 (100.00) | 9 (100.00) | 23 (100.00) | NA |
| CK (%) |  |  |  |  |
| (-) | 7 (20.59) | 3 (33.33) | 4 (16.00) | 0.5339 |
| (+) | 27 (79.41) | 6 (66.67) | 21 (84.00) |  |
| EMA (%) |  |  |  |  |
| (-) | 5 (14.71) | 2 (22.22) | 3 (12.00) | 0.8464 |
| (+) | 29 (85.29) | 7 (77.78) | 22 (88.00) |  |
| Desmin (%) |  |  |  |  |
| (-) | 28 (87.50) | 9 (100.00) | 19 (82.61) | 0.4575 |
| (+) | 4 (12.50) | 0 (0.00) | 4 (17.39) |  |
| WT_1 (%) |  |  |  |  |
| (-) | 11 (36.67) | 5 (62.50) | 6 (27.27) | 0.1795 |
| (+) | 19 (63.33) | 3 (37.50) | 16 (72.73) |  |
| Ki_67 |  |  |  |  |
| ≤60% | 21 (61.76) | 7 (77.78) | 14 (56.00) | 0.4515 |
| >60% | 13 (38.24) | 2 (22.22) | 11 (44.00) |  |
| myogenin (%) |  |  |  |  |
| (-) | 28 (96.55) | 7 (87.50) | 21 (100.00) | 0.6098 |
| (+) | 1 (3.45) | 1 (12.50) | 0 (0.00) |  |
| myoD1 (%) |  |  |  |  |
| (-) | 26(100.00) | 6 (100.00) | 20 (100.00) | NA |
| Bcl_2 (%) |  |  |  |  |
| (-) | 11 (40.74) | 4 (57.14) | 7 (35.00) | 0.5624 |
| (+) | 16 (59.26) | 3 (42.86) | 13 (65.00) |  |
| CyclinD1 (%) |  |  |  |  |
| (-) | 8 (33.33) | 1 (33.33) | 7 (33.33) | 1 |
| (+) | 16 (66.67) | 2 (66.67) | 14 (66.67) |  |
